# Supplementary material for: Evidence supported by Mendelian randomization: impact on inflammatory factors in knee osteoarthritis
Source: Front Med (Lausanne). 2024 May 28;11:1382836. doi: 10.3389/fmed.2024.1382836 (PMC11165061; doi:10.3389/fmed.2024.1382836)
Supplement: Supplementary file 1 [file Data_Sheet_1.ZIP › data/GCST90274809_5e6/809.leaveoneout.pdf]

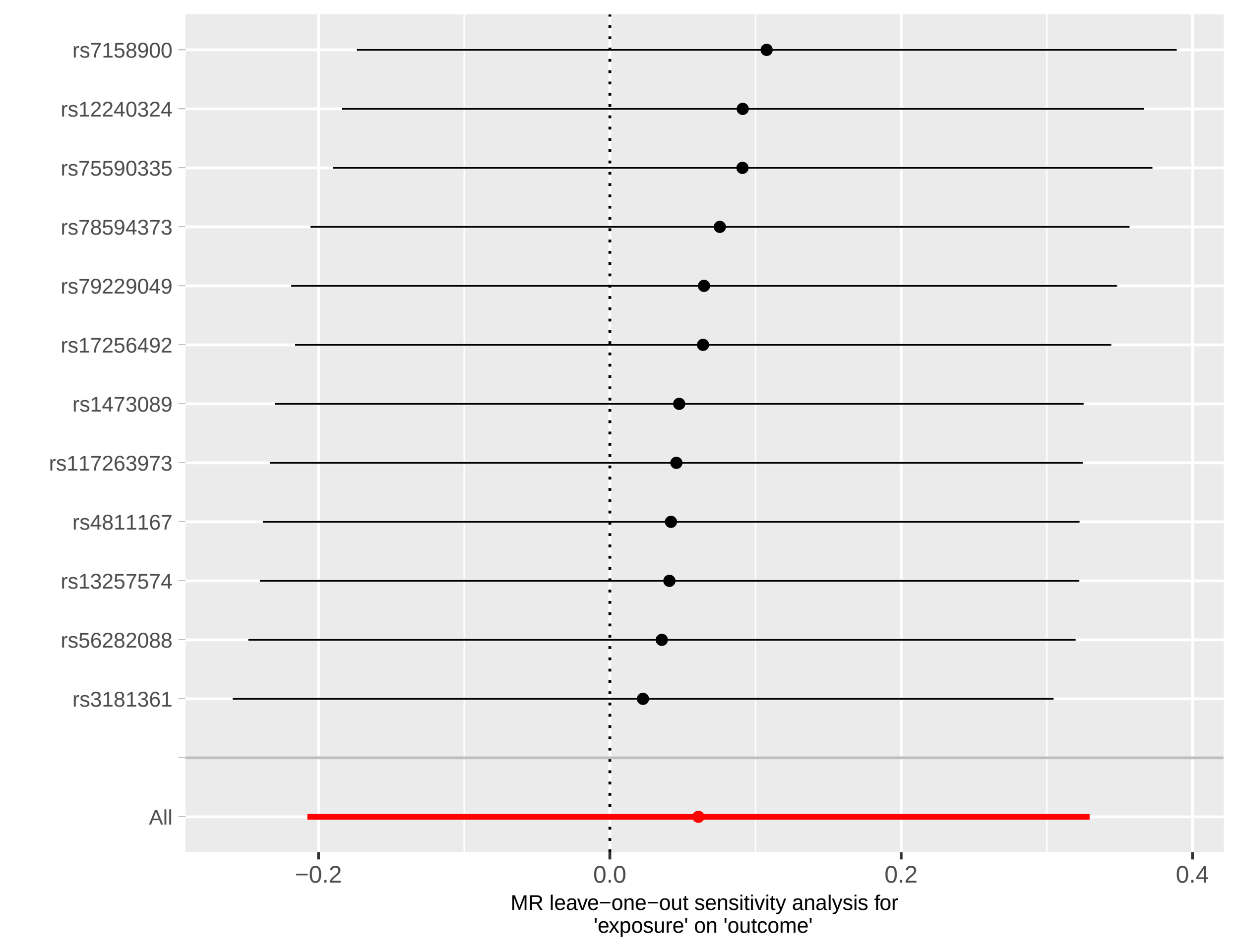
rs7158900
rs12240324
rs75590335
rs78594373
rs79229049
rs17256492
rs1473089
rs117263973
rs4811167
rs13257574
rs56282088
rs3181361
All
−0.2
0.0
0.2
0.4
MR leave−one−out sensitivity analysis for
'exposure' on 'outcome'
